# Robot-Assisted Radical Cystectomy: A Single-Center Experience and a Narrative Review of Recent Evidence

**DOI:** 10.3390/diagnostics13040714

**Published:** 2023-02-14

**Authors:** Bernardo Rocco, Giulia Garelli, Simone Assumma, Filippo Turri, Mattia Sangalli, Tommaso Calcagnile, Giorgia Gaia, Stefano Terzoni, Guglielmo Oliviero, Daniele Stroppa, Enrico Panio, Luca Sarchi, Alberto del Nero, Giorgio Bozzini, Angelica Grasso, Paolo Dell’Orto, Maria Chiara Sighinolfi

**Affiliations:** 1 Department of Urology, ASST Santi Paolo and Carlo, La Statale University of Milan, 20142 Milano, Italy; 2 Department of Obstetrics and Gynecology, ASST Santi Paolo and Carlo, La Statale University of Milan, 20142 Milano, Italy; 3 San Paolo Bachelor School of Nursing, ASST Santi Paolo and Carlo, 20142 Milano, Italy

**Keywords:** radical cystectomy, robotic surgery, intracorporeal urinary diversion, morbidity

## Abstract

Radical cystectomy represents one of the most challenging surgical procedures, exhibiting a high morbidity rate. The transition to minimally invasive surgery in the field has been steep, due to either the technical complexity and prior concerns of atypical recurrences and/or peritoneal spread. More recently, a larger series of RCTs has proven the oncological safety of robot-assisted radical cystectomy (RARC). Beyond survival outcomes, the comparison between RARC and open surgery in terms of peri-operative morbidity is still ongoing. We present a single-center experience of RARC with intracorporeal urinary diversion. Overall, 50% of patients had an intracorporeal neobladder reconstruction. The series confirms a low rate of complications (Clavien Dindo ≥ IIIa 7.5%) and wound infections (2.5%) and the absence of thromboembolic events. No atypical recurrences were found. To discuss these outcomes, we reviewed the literature related to RARC including level-1 evidence. PubMed and Web of Science searches were performed using the medical subject terms “robotic radical cystectomy” and “randomized controlled trial (RCT)”. Six unique RCTs comparing robot and open surgery were found. Two clinical trials dealt with RARC with an intracorporeal reconstruction of UD. Pertinent clinical outcomes are summarized and discussed. In conclusion, RARC is a complex but feasible procedure. The transition from extracorporeal urinary diversion (UD) to a complete intracorporeal reconstruction could be the key to improving peri-operative outcomes and reducing the whole morbidity of the procedure.

## 1. Introduction

Radical cystectomy (RC) represents the standard of care for very-high-risk non-muscle-invasive bladder cancer (NMIBC) and muscle-invasive non-metastatic bladder cancer (MIBC) [1]. As known, it is one of the utmost complex surgical procedures since it is made up of a dissection phase, including an extended nodal dissection, and a reconstructive step to restore the urinary tract. Even if bladder-sparing strategies are challenging the role of surgery [1,2], technological advances are making the surgical procedure as least invasive as possible. In the last decade, robotic surgery has gained a definite role in the treatment of bladder cancer: Its recognized advantages—magnification, 3D visualization, and the precision of dissection and suturing—seem to address the complexity of RC [2].

Nevertheless, the transition to robotic surgery for urothelial cancer has been steep, due to the prior concern of peritoneal seeding linked to minimally invasive surgery (MIS) [3,4]. In a review article including 31 studies, Mantica et al. found an incidence of 1.63% of atypical recurrences after a robot-assisted radical cystectomy (RARC), such as peritoneal carcinomatosis and port-site metastasis; however, the review was based on retrospective series and single reports [5]. Data on larger series demonstrated recurrence-free survival rates (RFS) of RARC similar to those reported for open surgery: this is also the case for the Mayo Clinic series, with a 5-year RFS of 70.8% and 64.7% for RARC and ORC, respectively [6]; or the 10-years’ experience from the International Robotic Cystectomy Consortium, stating that RARC was not associated with different patterns or higher relapse rates compared to historic ORC data [7,8]. To date, RFS, CSS, and OS of RARC have been documented similarly to those of ORC in all randomized controlled trials (RCTs), and the European Urological Guidelines suggest considering either the open or robotic approach for RC [2].

Beyond oncological safety, RARC remains a complex procedure, still displaying room for improvement in surgical strategies. The majority of surgical trials are based on the extracorporeal reconfiguration of urinary diversion (ECUD) through a mini-laparotomy; the evolution toward a total intracorporeal reconstruction of urinary diversion (ICUD) may be the cornerstone of maximizing the mini-invasiveness of the procedure.

The aim of this study is to report the single-center experience with RARC with ICUD to describe the surgical technique, diversion types, and early outcomes. Moreover, a narrative review of Level-1 evidence and best practices regarding RARC is provided to depict the ongoing transition from ECUD to ICUD.

## 2. Materials and Methods

This is a retrospective, single-center, observational cohort study about RARC performed at a high-volume robotic institution. Patients aged more than 18 years with histologically proven diagnoses of bladder cancer were included; MIBC and high-risk NMIBC with indication to radical cystectomy according to the EAU Guidelines and signed informed consent to the procedure were the inclusion criteria.

All patients were previously counseled on the intervention and the type of urinary diversion of choice; female patients received gynecological counseling too, to discuss the opportunity of a sexually sparing procedure. Male patients were counseled on the likelihood of a nerve-sparing RARC when deemed oncologically feasible.

### 2.1. Included Variables

A database was prospectively maintained and fulfilled by physicians not directly involved in the surgical team. The collected variables were:

- Demographics and pre-operative variables: Age, BMI, previous surgery, co-morbidities, ECOG, ASA score; cT, cN status; prior NAC.
- Intra-operative: Complication rate; urinary diversion rate stratified into ICUD (neobladder and ileal conduit), and uretero-cutaneostomy.
- Post-operative and pathological data: Histological type and differentiation; pT/ypT, pN/ypN; histological grade; surgical margin status; incidental finding of prostate cancer; length of stay (LOS); complication rate; Clavien–Dindo classification of complications; thromboembolic event, wound infection rate; 30-day overall re-admission rate.

### 2.2. Endpoint

In a center performing only complete intracorporeal procedures, the primary endpoint is to report the post-operative complication rate according to the Clavien–Dindo classification and the proportion of neobladder among the total number of RARC and the total number of ICUD.

Secondary endpoints were the intra-operative complication rate; thromboembolic event and wound infection rate; 30-day re-admission rate; CSS; and atypical recurrence in the case of availability of a 12 month follow-up.

### 2.3. Surgical Technique

The patient is placed in an 18° Trendelenburg position. The procedure starts with the identification and isolation of ureters bilaterally from above iliac vessels until bladder insertion. At the bladder level, the ureter is closed with a median size Hem-o-lok and then sectioned. In males, the peritoneum at the seminal vesicle (SV) level is incised and the plane between Denonvilliers’ fascia and the posterior face of the prostate is developed (between the bladder and vagina in females). Lateral aspects of the bladder are developed bilaterally, and vesical pedicles are clipped and transected. The access is facilitated by the use of the fourth arm to lift the bladder, which should be properly used especially in challenging cases with a high tumoral burden. In males, the preservation of the neurovascular bundle is performed when indicated. An inverse U peritonectomy is carried out between the 2 internal inguinal rings, umbilical arteries are transected, and access to the Retzius space is created. The anterior face of the bladder is developed, and the Santorini complex is severed and then sutured. The urethra is isolated and then incised after a large Hem-o-lok is placed to prevent urine spillage. The urethral stump is maintained for as long as possible. Frozen sections of distal ureters and urethra are performed; meanwhile, an extended pelvic nodal dissection is carried out bilaterally.

In women, the standard procedure includes removal of the bladder, the entire urethra, and adjacent vagina, uterus, distal ureters, and regional nodes; in the case of neobladder reconstruction, a pelvic-organ-preserving strategy is pursued, with preservation of the uterus and vagina to provide support to the reservoir. At this point, previous gynecological counseling is mandatory to evaluate gynecological history, sexual function, and possible prolapse.

An extended lymph node dissection (LND) was performed and included all nodes of the standard LND template, those in the region of the aortic bifurcation, presacral and common iliac vessels medial to the crossing ureters, the lateral borders of the genitofemoral nerves, the caudally circumflex iliac vein, the lacunar ligament, and the node of the Cloquet. In a single case, a super-extended LND template was used due to the extension of macroscopically involved nodes; in this case, LND extends cranially to the level of the inferior mesenteric artery.

**Figure 1:**
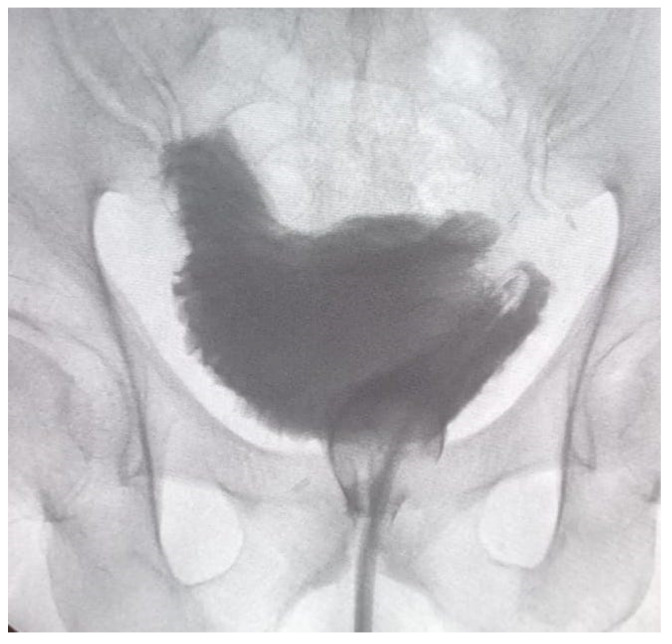
Orthotopic neobladder at a cystogram performed 18 days after surgery, before removing the urethral catheter.

In the case of neobladder reconstruction, the technique we used was that described by Asimakopoulos et al. [9]. A 40–50 cm ileal segment is isolated; the portion with a more adequate mesenteric length is chosen to be brought down to the pelvis. The median part of the isolated ileal segment is pushed towards the urethral stump, and the ileo-urethral approximation represents the first step. A modified posterior reconstruction is performed, with the first layer involving Denonvillier’s fascia and the rhabdosphincter and the second layer approximating the neobladder neck (created with a 1,5 cm ileal incision) and the urethral stump. Afterwards, the anastomosis is carried out; either the posterior reconstruction or ileo-urethral anastomosis is performed with a double-armed barbed suture (Stratafix3–0, Ethicon). The technique proceeds with the isolation of both ileal segments at each side using a mechanical laparoscopic stapler, and ileal-ileal anastomosis is accomplished. The reverse tubular U-segment of the ileum is detubularized to configure the neobladder. The reconstruction starts from the suture of the posterior plane, and then the cranial part is folded downwards toward the bladder neck to create the orthotopic reservoir with two lateral limbs. The neobladder is tested for leakage; uretero-neobladder anastomosis is then performed with direct anastomosis of each spatulated ureter in the dorsal part of the limbs (4–0 monocryl). Ureteral stents are placed before suturing the anterior plate and are brought out through the anterior abdominal wall. Figure 1 represents the cystogram of a neobladder before removing the trans-urethral catheter.

## 3. Results

The enrolment started on 15 July 2021 and ended on 2 November 2022. A total of 40 RARC were performed; the majority (36/40) were performed by a single surgeon (BR) and the remaining by two other surgeons (FT and MS), and two procedures were performed by visiting surgeons within a live surgery context. Table 1 reports demographic, pre-operative, and pathological data; Table 2 describes intra-operative and post-operative data, as well as complication rates. The mean age of patients was 66, and 82.5% were males. Twenty-three out of forty patients (57.5%) had a muscle-invasive disease at pathological histology; 22.5% (9/40) underwent a previous NAC. The wound infection rate was 2.5% (1/40); no thromboembolic events were evident. The overall rate of patients who received a neobladder was 50% (20/40); if considering the number of neobladders among patients who had an intestinal diversion, the neobladder rate was 68.9% (20/29); thus, the neobladder/ileal conduit ratio was approximately 2:1. The mean console time for RARC and neobladder reconstruction was 360 min (range: 210–480); the mean console time of RARC and nodal dissection alone was 180 min (range: 100–280). Median blood loss was 300 (IQR 100–450).

Three patients had a Clavien–Dindo complication ≥IIIa (7.5%). Clavien IIIa complications included two cases of lymphocele drainage and a single case of urinary leakage requiring nephrostomic tube placement; to note, two out of these three patients had a previous NAC, with the patient receiving nephrostomies displaying a C. Albicans superinfection. No spontaneous Clavien IIIb complication occurred. Two patients underwent a second procedure under general anesthesia due to unexpected consequences of post-operative delirium. In one case, the delirium occurred in a young adult male with previous drug abuse, who caused one of the ureteral catheters to re-enter the abdomen, thus a mini-laparotomy was required to retrieve the device; in the second case, post-operative disorientation led to the incidental dislodgement of the urethral catheter with bleeding, with the patient requiring an endourological revision and hemostasis of the neobladder.

Overall, 7/40 patients underwent post-operative adjuvant therapy (17.5%). Among patients with 12-month follow-ups, CSS was 92.8% (1/14). No atypical recurrence was recorded at any follow-up point.

## 4. Discussion

In a single-center prospective series, the use of robotic surgery for RC led to a 7.5% spontaneous complication rate, more or less equal to Clavien IIIa. From the literature, complication rate ≥IIIa in open surgery is approximately 16% [10,11]. Overall, 30/40 (75%) patients were out of the hospital after 30 days. No spontaneous Clavien III b complications occurred; as declared, two re-interventions were required due to episodes of post-operative delirium, one in a patient with prior drug abuse (dislocation of a ureteral stent) and the other a urethral catheter dislodgement in a neobladder patient who experienced post-operative delirium. The rate of wound infection was confirmed to be negligible (2.5%); thromboembolic events were completely absent in the present cohort, with 30% of patients having >75 years. Two out of three patients with a Clavien >IIIa complication had received a previous NAC; consistently, Hussein et al. found that NAC is a risk factor for 90-day re-admission (OR 2.2, 95%CI 1.15–4.31, *p* = 0.02) [7].

Unlike other studies, the use of neobladder in the whole RARC cohort was high (50%) [10]; when considering only ICUD (neobladder and ileal conduit), the proportion of neobladder reconstruction was even higher (68.9%). In the article by Catto et al., the rate of intracorporeal reconstruction of a neobladder was 12% [10]; Hussein et al. reported a rate of 9% of the intracorporeal neobladder within the multicentric database of the International Robotic Cystectomy Consortium [7]. In our series, we opted to pursue the neobladder as a first-choice diversion, whenever deemed appropriate and discussed with the patient; we found similar operative time between neobladder and ileal conduit configuration, without differences in post-operative complications (data not presented). To note, all patients had prior comprehensive counseling with the surgical team, which led to the absence of post-operative regret about the type of diversion chosen. It is well known that after the intervention, the primary issue of the patient is related to cancer treatment; thereafter, patient priorities focus on regaining a sense of normalcy and quality of life, thus the recovery of better body image may support a better quality of life with an orthotopic neobladder [12].

From the present series, robotic surgery with ICUD provides a low rate of post-operative morbidity, confirming outcomes from most recent RCTs.

To date, six RCTs have been published on RARC compared to open surgery; further analysis of secondary endpoints was delivered from the same cohorts.

The very preliminary studies focused on oncological issues and evaluated the safety of MIS in the field of urothelial cancer. One of the most robust was the RAZOR trial (Randomized Open versus Robotic Cystectomy, 2018), which stated the non-inferiority of RARC compared to the open approach in terms of the 2-year progression-free survival and other oncological outcomes [13]. By including only extracorporeal diversion reconstruction, the study failed to demonstrate a clear advantage of robotic over open RC in terms of post-operative morbidity. An update of the RAZOR trial was published in 2020 and analyzed QoL items through the FACT-Vanderbilt Cystectomy index subscale and the Short Form 8 Health Survey (SF-8); once more, the trial showed the lack of any significant difference in health-related QoL in robotic and open cystectomies [14]. A previous RCT by Nix et al. randomized 41 patients to receive RARC with ECUD or ORC and focused on the nodal yield as the primary endpoint. Beyond stating non-inferiority of the primary endpoint, the trial reported similar outcomes in terms of the overall complication rate and LOS [15]. Another RCT published in 2016 by Bochner et al. failed to identify an advantage for robot-assisted techniques for patients undergoing RC [16]. At 90 days, Clavien–Dindo grade 2–5 complications were similar between ORC and RARC (66% vs. 62%, *p* = 0.7) as well as LOS—8 days in both arms—and 3- and 6-month QoL outcomes [16]. Early outcomes from another RCT (CORAL trial, which enrolled 60 patients randomized to RARC, ORC, and laparoscopic RC, LRC) concluded the superiority of LRC in terms of post-operative course (30-day complication rates of 55%, 26%, and 70% for RARC, LC, and ORC, respectively) [17]; the 90-day complication rate and long-term oncological outcomes were similar between groups (5-year RFS of 58%, 71%, and 60% and the CSS was 68%, 69%, and 64%, for RARC, LRC, and ORC, respectively) [18].

To note, all the aforementioned RCTs used an extracorporeal reconfiguration of urinary diversion and failed to demonstrate a clear benefit of robotic surgery in terms of post-operative morbidity. It has been argued that a mini-laparotomic approach with ECUD may mitigate the advantages of robotic surgery, i.e., the absence of peritoneum exposure and improved tissue handling.

More recently, RCTs comparing ORC and RARC with a complete intracorporeal approach have been published. Mastroianni et al. designed an RCT to demonstrate the superiority of RARC with intracorporeal UD in terms of a 50% transfusion rate reduction [11]. By randomizing 116 patients (58 RARC, 58 open surgeries), overall peri-operative transfusion rates were significantly lower in the RARC cohort (22%) compared to the open one (41%). When addressing patient-related QoL on a subset of patients with a 1-year follow-up, the equivalence between approaches for most QoL domains was evident [19]. Another recent RCT comparing peri-and post-operative outcomes of robotic and open surgery for RC was recently released, namely, the iROC trial [10]. By including 317 patients who underwent RC with robot-assisted or open surgery, those with RARC with complete intracorporeal UD had an advantage in terms of the primary outcome—the number of days alive and out of the hospital within 90 days of surgery; moreover, when investigating secondary endpoints, RARC with intracorporeal UD was also able to reduce thromboembolic complications (1.9% vs. 8.3%) and wound complications (5.6% vs. 16.0%). The QoL items were better for RARC patients at 5 weeks, whereas disability scores—investigated using the World Health Organization Disability Assessment Schedule 2.0—were improved at 5 and 12 weeks within the robotic arm. Cancer recurrence and overall mortality were similar between groups [10]. The results of these RCTs are consistent with those we reported, and the use of complete ICUD could be the key to maximizing the mini-invasiveness of RARC. A summary of all RCT findings is reported in Table 3.

The present study is not devoid of limitations. First, the small sample size and the single-center feature may limit the generalizability of the results. Second, the previous high robotic expertise of the first surgeon—the one who performed the majority of the procedure (90%)—may represent a further bias. However, it should be noted that the current series was performed in a center previously naïve to robotic urological surgery. Thus, the previous expertise of the robotic team may have supported the safe introduction of RARC even inside a center without experience in minimally invasive surgery.

In conclusion, RARC with ICUD may be considered a safe and effective option for the treatment of bladder cancer. Level-1 evidence from the literature confirmed the safety of robotic surgery by ruling out the risk of increased cancer recurrence rates or atypical recurrences. The superiority of the robotic approach in terms of post-operative morbidity was definitively described in two RCTs published in 2022 that used the intracorporeal reconfiguration of UD.

## Figures and Tables

**Table 1:** Demographic, pre-operative, and final pathological data.

Age, Mean (SD), Range	66 (10), 46–80
Sex	
Male	33/40 (82.5)
Female	7/40 (17.5)
Eastern Cooperative Oncology Group Performance Status	
0: Fully active	32/40 (80%)
1: Restricted in strenuous activity	5/40 (12.5)
2: Self-caring but unable to work	1/40 (2.5)
3: limited self-care	1/40 (2.5)
BMI	26 (3.7), 17–37
Smoking (current or previous) (%)	25/40 (62.5)
Preoperative hemoglobin, mean (SD), g/dL	12.8 (1.8), 9–16
Preoperative creatinine, mean (SD), ml/dL	1 (0.2), 0.6–1.7
Neoadjuvant chemotherapy	9/40 (22.5%)
Cystectomy histology pathologic tumor stage	
pT0 or ypT0	6 (15)
pTa	2 (5)
pTis	6 (15)
pT1	3 (7.5%)
pT2	11 (27.5%)
pT3	7 (17.5)
pT4	5 (12.5)
Cystectomy Histology Grade	
Low (LG)	5/34 (15%)
High (HG)	29/34 (85%)
Positive surgical margins	0
Histological stage N	
N0 (%)	29 (72.5)
N1 (%)	8 (20)
N3 (%)	1 (2.5)
N3 (%)	1 (2.5)
Concomitant Prostate cancer (%)	6/33 (18.1)

**Table 2:** Intra-operative and post-operative data and complication rates.

Type of Diversions	
Intracorporeal neobladder	20 (50)
Ileal conduit	9 (22.5)
Ureterocutaneostomy	11 (27.5)
Intra-operative complications	1/40 (2.5)
Post-operative complications—Clavien-Dindo Classification	
I (%)	8/40 (20)
II (%)	11/40 (27.5)
IIIa (%)	3/40 (7.5)
IIIb (%)	0/40 *
IV (%)	0
Thromboembolic event	0
Wound infection	1/40 (2.5)
30-day re-admission rate	
No	30 (75)
Yes	10 (25)

* No spontaneous Clavien IIIb complications occurred. Two patients underwent a second procedure under general anesthesia due to unexpected consequences of post-operative delirium.

**Table 3:** Summary of findings from RCTs comparing surgical approaches.

Author	Years	Comparison	N° of Patients	Primary End Point	Secondary End Point	ICUD/ECUD	Main Findings
Parekh et al. (RAZOR) [13]	2018	ORC vs. RARC	302	2-year progression-free survival	Adverse events Urinary tract infection Postoperative ileus	ECUD	2-year PFS 72.3% (95% CI 64.3 to 78.8) with RARC 2-year PFS 71.6% (95% CI 63.6 to 78·2) with ORC (difference of 0.7%, 95% CI −9.6% to 10.9%; *p* non-inferiority = 0.001) Adverse events: 101 (67%) of 150 RARC; 105 (69%) of 152 ORC
Becerra et al. (RAZOR update) [14]	European urology 2020	ORC vs. RARC		Quality-of-care indicators (QOCIs)		ECUD	No difference
Nix et al. [15]	European Urology 2009	ORC vs. RARC	41	Primary end point: LN yield.	Perioperative outcomes: EBL, OR time, Time to flatus, Time to BM, Length of stay In-house analgesia, Clavien complication	ECUD	LN—non inferiority Results favor RARC in several perioperative parameters including EBL and narcotic requirements; longer OT
Bochner et al. [16]	European Urology 2014	Open vs. RARC	118	90-d grade 2–5 complications	comparison of high-grade complications, EBL, OT, pathologic outcomes, PSM, 3- and 6-month patient-reported quality-of-life (QOL), and total operative room and inpatient costs.	ECUD	At 90 d, grade 2–5 complications were 62% with RARC and 66% ORC (95% CI for difference, 21% to 13%; *p* = 0.7). The RARC group had lower EBL (*p* = 0.027) but significantly longer OT than the ORC. Margins and lymph node yields were similar; LOS was 8 d in both arms Three- and six-month QOL outcomes were similar between arms. Cost analysis demonstrated an advantage for ORC compared with RARC.
Khan et al. (CORAL) [17]	2015	Open vs. RARC vs. LRC	60	30- and 90-d complication rates.	perioperative clinical, pathologic, and oncologic outcomes, and quality of life (QoL)	ECUD	30-d complication rates (ORC: 70%; RARC: 55%; LRC: 26%; *p* = 0.024). The 90-day rate did not differ (ORC: 70%; RARC: 55%; LRC 32%; *p* = 0.068). Mean OT was significantly longer in RARC compared to ORC or LRC. There were no significant differences in QoL
Khan et al. (CORAL) [18]	2020	Open/LPS/RA	60	5-year oncological outcomes: Recurrence-free survival (RFS), cancer-specific survival (CSS), and overall survival (OS).		ECUD	The 5-year: RFS was 60%, 58%, and 71%; CSS was 64%, 68%, and 69%; OS was 55%, 65%, and 61% for ORC, RARC, and LRC No significant differences
Catto et al. (iROC) [10]	2022	ORC vs. RARC	317	recovery and morbidity— number of days alive and out of the hospital within 90 days of surgery.	20 secondary outcomes, including complications, quality of life, disability, activity levels, and survival	ICUD	82 vs. 80 days alive and out of the hospital within 90 days of surgery; the clinical importance of these findings remains uncertain; advantage of RARC in terms of wound infection and thromboembolic event
Mastroianni et al. [11]	2022	ORC vs. RARC	116	To evaluate the superiority of RARC with ICUD in terms of 50% transfusion rate reduction	Early outcomes	ICUD	22% and 44% peri-operative transfusion rates with RARC and ORC, confirming a benefit for RARC with ICUD; peri-operative complications, LOS and 6-mo QoL similar between groups
Mastroianni et al. [19]	2022	ORC vs. RARC	51	1-year health-related quality of life (HRQoL) questionnaires Global health status/QoL Physical functioning Emotional functioning Social functioning Fatigue Pain Insomnia Constipation sexual functioning, urinary symptoms, abdominal bloating and flatulence, diarrhea, appetite loss, dyspnea, nausea and vomiting,	Perioperative and early postoperative outcomes, EBL, ERAS protocol, hospital stay, perioperative complication, readmission 30–90 days, complications at 30 days and 90 days.	ICUD	Both groups significant worsening of body image and physical and sexual functions (all *p* 0.012). Patients receiving ORC were more likely to report significant 1-year impairment of role functioning, symptoms scales and bowel symptoms (all *p* 0.048). Patients receiving RARC reported significant impairment of urinary symptoms and problems (*p* = 0.018) Robotic surgery seems to provide benefits for most quality-of-life items on patient

## Data Availability

The data presented in this study are not publicly available due to privacy restrictions.
